# Advance care planning for patients with cancer and family caregivers in Indonesia: a qualitative study

**DOI:** 10.1186/s12904-022-01086-0

**Published:** 2022-11-22

**Authors:** Diah Martina, Christina Yeni Kustanti, Rahajeng Dewantari, Noorwati Sutandyo, Rudi Putranto, Hamzah Shatri, Christantie Effendy, Agnes van der Heide, Carin C. D. van der Rijt, Judith A. C. Rietjens

**Affiliations:** 1 Department of Medical Oncology, Erasmus MC Cancer Institute, University Medical Center Rotterdam, P.O. Box 2040, 3000 CA Rotterdam, the Netherlands; 2 Department of Public Health, Erasmus MC, University Medical Center Rotterdam, Rotterdam, the Netherlands; 3 Division of Psychosomatic and Palliative Medicine, Department of Internal Medicine, Faculty of Medicine Universitas Indonesia, Jakarta, Indonesia; 4 Cipto Mangunkusumo National General Hospital, Jakarta, Indonesia; 5 Sekolah Tinggi Ilmu Kesehatan Bethesda Yakkum, Yogyakarta, Indonesia; 6 Department of Neuro-Psychiatry, Dharmais National Cancer Center, Jakarta, Indonesia; 7 Department of Hematology and Medical Oncology, Dharmais National Cancer Center, Jakarta, Indonesia; 8 School of Nursing, Faculty of Medicine, Public Health and Nursing, Universitas Gadjah Mada, Yogyakarta, Indonesia

**Keywords:** Advance care planning, Patient, Cancer, Family, Collectivist, Religiosity, Asia, Indonesia

## Abstract

**Background:**

Individuals’ willingness to engage in advance care planning is influenced by factors such as culture and religious beliefs. While most studies on advance care planning in Asia have been performed in high-income countries, Indonesia is a lower-middle-income country, with a majority of strongly collectivist and religiously devout inhabitants. We studied the perspectives of Indonesian patients with cancer and family caregivers regarding advance care planning by first exploring their experiences with medical information-disclosure, decision-making, and advance care planning and how these experiences influence their perspectives on advance care planning.

**Methods:**

We conducted semi-structured interviews among 16 patients with cancer and 15 family caregivers in a national cancer center in Jakarta and a tertiary academic general hospital in Yogyakarta. We performed an inductive thematic analysis using open, axial, and selective coding. The rigor of the study was enhanced by reflective journaling, dual coding, and investigator triangulation.

**Results:**

Twenty-six of 31 participants were younger than 60 years old, 20 were Muslim and Javanese, and 17 were college or university graduates. Four major themes emerged as important in advance care planning: (1) participants’ perceptions on the importance or harmfulness of cancer-related information, (2) the importance of communicating bad news sensitively (through empathetic, implicit, and mediated communication), (3) participants’ motives for participating in medical decision-making (decision-making seen as patients’ right or responsibility, or patients’ state of dependency on others), and (4) the complexities of future planning (e.g., due to its irrelevance to participants’ religious beliefs and/or their difficulties in seeing the relevance of future planning).

**Conclusions:**

Culturally sensitive approaches to advance care planning in Indonesia should address the importance of facilitating open communication between patients and their families, and the various perspectives on information provision, bad news communication, and decision-making. Advance care planning should focus on the exploration of patients’ values, rather than drafting treatment plans in advance.

**Supplementary Information:**

The online version contains supplementary material available at 10.1186/s12904-022-01086-0.

## Background

Advance care planning is a process of defining and discussing values, goals and preferences for future medical treatment and care [1]. It is increasingly seen as an essential element of high-quality end-of-life care. However, as the concept is rooted in the Western philosophy of person-centered care and self-determination, it may not always be relevant in countries where the cultures favor collectivism and the maintenance of social harmony over individual autonomy [2–4]. Our recent systematic reviews of studies from southern, south-eastern, and eastern Asian countries showed that proper understanding of one’s illness (including its prognosis) is regarded as an important initial step towards engagement in advance care planning [5]. The uptake of advance care planning is further influenced by patients’ beliefs and healthcare professionals’ fear of creating conflict with family members [5–7]. Few studies provided in-depth insight into patients’ and families’ perspectives on advance care planning, and few were conducted in low and middle-income Asian countries, including Indonesia [5–7].

Indonesia is the fourth most populous country in the world, with the prevalence of cancer increasing from 1.4 per 1000 people in 2013 to 1.8 per 1000 in 2018 [8]. In 70% of these patients, the illness is at an advanced stage [8], where advance care planning may have added value. Although a survey among participants from a general population in Indonesia showed that the majority wished to be informed about a possible life threatening disease and be engaged in end-of-life communication, a study on the perspectives of Indonesian patients has not been performed [9]. However, the stigma surrounding cancer prevented people to have an open communication about it [10]. In addition to that, Indonesia not only follows Asian traditions of family-centeredness in medical decision-making, it is also one of the most religious countries in the world where the majority of its population consider religious values to be important to their lives [11–13]. These factors may all influence people’s perspectives on advance care planning and their willingness to engage in it [5].

To better understand the possible value of advance care planning for cancer patients in Indonesia, we aimed to provide in-depth insight into the perspectives of patients with cancer and family caregivers. To facilitate the exploration of participants’ perspectives in advance care planning, we first explored their experiences with medical information-disclosure, decision-making, and advance care planning before exploring how these experiences influence their perspectives on advance care planning.

## Methods

### Study design

This exploratory qualitative study involved in-depth interviews with patients with cancer and family caregivers. We performed inductive thematic analysis using open, axial, and selective coding [14, 15]. Firstly, we facilitated participants’ self-conscious reflection of their experience with living with cancer, particularly with medical information disclosure, decision-making, and advance care planning. We further explore participants’ perspectives on advance care planning drawn from their reflection and meaning-making of these experiences [16, 17].Reporting was guided by the Consolidated Criteria for Reporting Qualitative Research (COREQ) [18].

### Study setting

The study was conducted in an Indonesian national cancer centre in Jakarta and a tertiary academic general hospital in Yogyakarta.

### Sampling and recruitment

Oncologists in participating wards selected patients with cancer who were at least 18 years of age, spoke Indonesian, had been diagnosed with cancer for at least 6 months, were aware of their diagnosis, and agreed to participate in the study. The oncologists also selected family caregivers of patients with cancer who were also at least 18 years of age, spoke Indonesian, were the primary caregiver for the patient, and agreed to participate in the study. These participants were purposively sampled to capture the diversity of their demographic characteristics (age, sex, cancer diagnosis, education).

### In-depth interviews and data collection

Semi-structured in-depth interviews were conducted face-to-face from July to September 2019 by DM, a female Indonesian physician specializing in internal medicine and palliative care, who was also trained to perform qualitative studies, and CYK, a female Indonesian nurse and researcher, trained and experienced in qualitative studies. RD, a female Indonesian physician, who specialized in psychiatry and palliative care and was also experienced in qualitative studies, made additional notes based on her observations during the interviews.

We developed a topic guide for the interviews based on our systematic reviews of advance care planning in Asia [5, 6] and consultations with various experts in medical oncology, palliative care, psychosomatic medicine, psychology, and research. The interview guide (Appendix 1 and 2) contained an introduction to the study and to advance care planning and was designed to elicit (1) participants’ experience and preferences regarding information provision; (2) their values and preferences regarding current and future care; (3) their experience with and perspectives on advance care planning; and (4) their perspectives on their role in advance care planning.

As the concept of advance care planning is unfamiliar in Indonesia, there is no Indonesian term for it. In this study, we therefore used the international consensus definition of the European Association for Palliative Care: “advance care planning enables individuals with the decisional capacity to identify their values, to reflect upon the meanings and consequences of serious illness scenarios, to define goals and preferences for future medical treatment and care, to discuss these with family and healthcare professionals, and to record and review these preferences if appropriate.” [1]

### Data processing and analysis

All interviews were audio-recorded and transcribed verbatim in Indonesian (the official language at the study sites) by DM and CYK. We conducted an inductive thematic analysis using open, axial, and selective coding of these interview transcripts and the field notes. We followed six phases of thematic analysis by Braun & Clarke (Appendix 3) [14, 15]. Before identifying ideas, two coders (DM and CYK) familiarized themselves with the data by reading all of the transcripts several times (Phase-1). After DM and CYK gained trustworthy familiarity and captured the core meaning of the empirical materials, they selected four transcripts (two transcripts of patients interviews and two of family caregiver interviews) on the basis of their richness – e.g., the transcripts that reveal the complexities and the richness of the topic that is being studied [19]. Independently, they then generated initial codes (open coding) of the transcripts (Phase-2). Afterwards, they independently grouped the codes under broader themes (Phase-3). To achieve consensus, codes and themes were discussed several times between DM and CYK.

**Fig. 1:**
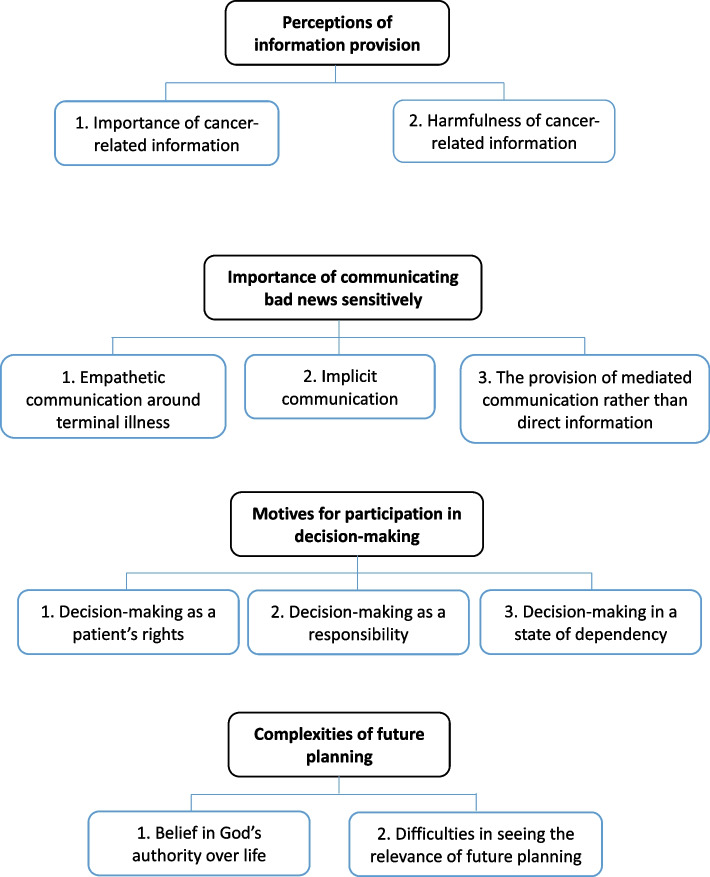
Coding tree of the perspectives on serious illness communication

To enhance the validity of the findings, we performed investigator triangulation, where two or more researchers were involved in observing and generating a conclusion (Phase-4). For this purpose, the four selected transcripts were then translated into English by a professional translator and shared with JR, AH, and CR (the non-Indonesian-speaking investigators). This process allowed the non-Indonesian-speaking investigators to gain familiarity with the materials [19]. Likewise, codes and themes were translated into English to facilitate the discussions. Codes, themes, quotes, and empirical materials (Indonesian transcripts of all interviews) were also shared with bilingual co-investigators (CE, RP, HS, RD, NS). Finally, codes and themes were discussed with members of the research team, with backgrounds in oncology, nursing, psychiatry, epidemiology, health sciences, palliative medicine, and psychosomatic medicine. The open codes were then organised into an initial coding tree, by going back and forth through the themes and the transcripts, using the constant comparative method (axial coding). The initial coding tree that had been discussed with the team members was tested by DM and CYK on another four transcripts. These newly developed codes were discussed with the larger team, and the coding tree was adjusted accordingly. This process was completed when all transcripts had been coded and the final coding tree had been developed. Members of the research team met several times to refine this final coding tree (Phase-5), by selecting core concepts, systematically connecting these core concepts with other categories, and filling in the categories that need to be refined (selective coding). All steps were iterative and reflective, developing over time and involving a constant moving back and forward between phases. Finally, all investigators were involved in the writing of the manuscript (Phase-6). Qualitative data analysis software (N-Vivo version 12) was used to assist in data analysis.

The qualitative rigor of the study was enhanced through the stimulation of credibility, confirmability, reflexivity, and transferability. Credibility was stimulated through investigator triangulation and data source triangulation where we explored various participants’ perspectives: patients with cancer and family caregivers. Confirmability and reflexivity were stimulated through reflective journaling by dual coders to enable reflection on the findings as well as their own emotions during the interviews. In addition, during regular meetings with team members, reflective journaling was used to discuss interviewers’ and researchers’ potential biases and subjectivities to the studied topic and how these might affect their interpretations. Transferability was stimulated through ‘thick description’ – a rich account of descriptive data including the context in which the research was carried out – of the participants and the research process (setting, sample, sample size, sample strategy, demographic and clinical characteristics, and interview guide) to enable the reader to assess whether our findings are transferable to their own settings.

## Results

### Participants’ demographics

We interviewed 16 patients and 15 family caregivers from unrelated families. All of the participants that were approached agreed to participate in the study. Each interview lasted approximately 45 minutes. After the analysis of the last interviews (with a patient and with a family caregiver), we did not find new themes, and therefore we concluded to have reached data saturation.

Table 1 summarizes the participants’ characteristics. Eight of the 15 family-caregiver participants were spouses, and 26 of the 31 participants were younger than 60 years old, 20 were Muslims and Javanese, and 17 were college or university graduates.

**Table 1:** Participants’ demographic characteristics

	Patients (***N*** = 16)	Family caregivers (***N*** = 15)
**Sex**		
Male	7	9
Female	9	6
**Age (years)**		
< 40	6	4
40–60	7	9
> 60	3	2
**Types of cancer**^a^		
Blood cancer	5	3
Lung cancer	1	2
Gastrointestinal cancer	2	2
Breast cancer	6	4
Cervical cancer	2	4
**Stage**		
I	1	1
II	3	4
III	5	3
IV	4	5
No stage (Leukaemia)	3	2
**Education**		
No formal education	1	0
Elementary school	1	1
High school	5	6
College/university	9	8
**Relationship with patient**		
Spouse	–	8
Parent	–	0
Child	–	5
Daughter/son-in law	–	2
Religious affiliation		
Islam	10	10
Catholic	2	4
Christian	4	1
Race		
Javanese	12	8
Sundanese	2	5
Chinese	–	1
Batak	1	–
Malay	1	–
Minangkabau	–	1

^a^Cancer diagnosis of the patient participants or of the relatives of family caregiver participants

### Thematic findings

Four main themes were identified as key features of the perspectives on serious illness communication (Fig. 1): 1) perceptions of information provision; 2) importance of communicating bad news sensitively; 3) motives for participation in decision-making; and 4) complexities of future planning.

#### Theme-1: perceptions of information provision

Our study showed that participants’ wishes for cancer-related information were influenced by their perceptions of (a) whether the information was important or relevant to them, and (b) whether they considered the information harmful.

### Importance of cancer-related information

Many patient participants wished to receive information about their illness, particularly their diagnosis, treatment options, and, to a certain extent, their prognosis. They considered such information important because it would foster their autonomy in further decision-making.

*“So I’ll know what will be the next [step] is, and so I won’t have to depend on my children, right? They have their own jobs and live far away… I must know, so I’ll have no regret in the future.”* (YK3A: female patient (age 62) with stage III breast cancer, Christian, Javanese.)

Likewise, some family caregiver participants who acknowledged the patients’ main role in decision-making thought it was important that provision of information is guided by patients’ needs.

While the information was often delivered to the patient through family members, those who considered it to be the patients’ right to receive information indicated that they would support truth-telling.

*“At the beginning, I was the only one who knew [about the patient’s illness]. But as time went by, I asked the doctor ‘Doc, I’d like your help in explaining my husband’s illness to him. I don’t want to lie to him. He has the right to know.´ That’s what I said to the doctor.”* (RSKD3B: wife (age 47) of a patient with stage IV lung cancer, Muslim, Sundanese.)

Some family caregiver participants believed that providing patients with medical information is necessary to maintain trust within the family.

*“If my wife doesn’t know and later she would find out from somebody else, it could be serious. She might think that we [as her family] had not been open [with her].”* (YK3B: husband (age 49) of a patient with stage II cervical cancer, Muslim, Javanese.)

Nevertheless, while most patient participants considered truth-telling important, some patients and family caregivers regarded certain information as irrelevant, particularly information on estimated life expectancy, due to their belief that death is unpredictable or predetermined by God.

*“As for myself, I don’t need a number (for life expectancy) because, once we know that we’re ill [i.e., have been diagnosed with cancer], the number is unnecessary. It (death) can happen anytime.”* (RSKD9A: female patient (age 32) with acute lymphocytic leukaemia, Islam, Javanese.)

### Harmfulness of cancer-related information

Participants who believed that certain information could harm their loved ones indicated that they would conceal such information to protect their loved one’s psychological wellbeing.

Patients and family caregivers alike said they would conceal information: patient participants would conceal “harmful” information to their family members and vice versa. Withholding burdensome information was commonly considered to be an act of love.

*“I would feel sorry for my family [if they knew about my poor prognosis]. Let me bear the burden myself.”* (YK6A: female patient (age 67) with stage II lung cancer, Christian, Javanese.)

*“If my mother were present [when bad news was communicated], it would burden her thoughts. It would be enough to discuss the more detailed and deeper information with me. My mother doesn’t need to know. [I believe that] one’s thoughts can influence one’s condition.”* (YK2B: daughter (age 39) of a patient with stage II cervical cancer, Christian, Javanese.)

Some family caregiver participants believed it was their duty to preserve patients’ hope.

*“Family members are the ones who should encourage and keep the patient’s spirits up … Mom knows about her illness, but the full risk [of death] – we don’t have the heart [to tell her].”* (YK1B: Son (age 34) of a patient with advanced non-Hodgkin lymphoma, Muslim, Javanese.)

### Theme-2: importance of communicating bad news sensitively

Patient and family caregiver participants expected empathetic communication with their preferences for how information should be delivered being taken into account. Overall, participants mentioned three preferred ways for the delivery of bad news, namely: (a) through empathetic communication around terminal illness; (b) through implicit (i.e. indirect or euphemistic) communication; or (c) through mediated rather than direct truth-telling.

### Empathetic communication around terminal illness

Patient and family caregiver participants considered it important to approach the communication of bad news surrounding terminal illness empathetically. Communication that takes away hope (e.g. hope for cure) is not considered empathetic

*“It actually depends on how it is communicated. Sometimes, for example, a doctor said, ‘This is already severe; it can’t be treated any more’. It shouldn’t be presented like that, right? But I’m sorry, sometimes it happens.”* (YK5A: female patient (age 48) with recurrent metastatic breast cancer, Muslim, Javanese.)

Similarly, communication that created a sense of abandonment was not seen as empathetic communication either.

*“If you’d heard the doctor’s statement when he gave up, you’d have been shocked, because he said ‘oh, that’s how it is, let’s hand it to God and hope for a miracle’ in front of the patient and family, and also the nurses. People were speechless! How can a doctor say anything like that?”* (YK4B: husband (age 51) of a patient with stage IV breast cancer, Catholic, Javanese.)

### Implicit communication

Throughout the interviews, many patient and family caregiver participants used implicit formulations (euphemisms) to avoid direct communication, saying for example “illness” rather than “cancer”; “it” or “leave” rather than “death;” and “serious” rather than “malignant”. Accordingly, as they considered the use of direct words to be blunt, they appreciated communication that was more euphemistic.

*“So the [doctor’s] communication was very pleasant. I mean, not too serious – quite relaxed. When the pathology results came in, the doctor told me, not that it was malignant, only that in the next hospital I may receive chemo or radiation according to what they would conclude there. [The doctor said] ‘The most important thing is that you keep the spirit, eat a lot, take good care of your condition’.”* (YK7A: female patient (age 45) with cervical cancer stage IIB, Muslim, Javanese.)

### The provision of mediated communication rather than direct information

Some participants considered conveying bad news through family members a sensitive approch. One patient participant felt that information about life expectancy could best be delivered through family members. She believed that her family members could better judge than healthcare professionals whether such information was necessary because they knew her personality.

*“It [life expectancy] needs to be communicated, but not to the patient – there has to be a mediator for that. And it is up to the family whether they want to deliver it to the patient or not.”* (RSKD9A: female patient (age 32) with acute lymphocytic leukaemia, Muslim, Javanese.)

Several family caregiver participants believed they could convey sensitive information better than healthcare professionals as they would be able to minimize its harm to patients’ mental wellbeing. They believed that, given their longer and closer relationship with the patient, they knew the best approach and timing for conveying such information.

*“Every family has its own communication techniques. Once, I took over the conversation because the doctor was too spontaneous, bla, bla, bla, as is. I just followed. Mmm … we’ve often seen on television that there’s always a separate communication between family and patient [after the communication between the doctor and the family]. It should be like that, not too vulgar, though afterwards, the patient must still know about her condition.”* (YK4B: husband (age 51) of a patient with stage IV breast cancer, Catholic, Javanese.)

Nevertheless, some patient participants reported their preference for direct, non-mediated communication with healthcare professionals.

*“I must discuss it with my family, though I’ll be the one who talks [to the physician].”* (RSKD9A: female patient (age 32) with acute lymphocytic leukaemia, Muslim, Javanese.)

#### Theme-3: motives for participation in decision-making

Our study showed that participants’ preferences for involvement in decision-making ranged from a patient-centered style, through a family-led style, to a physician-led (paternalistic) style.

These preferences were influenced by: (a) whether patients considered involvement in decision-making to be a patient’s right; (b) whether they believed patients should be given the opportunity to take control of their care; (c) whether they considered it as patients’ or family caregiver’s responsibility; and (d) whether they were in a state of dependency regarding decision-making. Regardless of their motives, many patient participants greatly valued family involvement and a guiding role from physicians.

### Decision-making as a patient’s right

Patient participants who considered decision-making their right were likely to take an active role while still seeing family involvement as essential.

*“As long as I can still take a role [in decision-making], then I will. Unless my condition is already … when I can only lie down or am unconscious … then maybe somebody else can take the decision. My Mom or someone else. But as long as I still have the right to do it and am still capable of doing it, then I’ll do it.”* (RSKD9A: female patient (age 32) with acute lymphocytic leukaemia, Muslim, Javanese.)

Some participants considered that patients’ involvement in decision-making was an opportunity for patients to take control of their care, even when seeing their family’s involvement as important.

*“Actually, I would like to communicate the options with my family. Although their opinions may differ from mine, I will be the one who eventually decides. The most important thing is that, later, I will have no regrets.”* (YK6A: female patient (age 67) female patient with stage II lung cancer, Christian, Javanese.)

Likewise, family caregiver participants who agreed that decision-making is a patient’s right were likely to acknowledge and respect the patient’s leading role in it.

*“Everyone [in the family] would be invited to join the discussion, but the patient will make the final decision. We only provide her with considerations.”* (YK4B: husband (age 51) of a patient with stage IV breast cancer, Catholic, Javanese)

### Decision-making as a responsibility

While some patient participants considered decision-making – and its possible consequences – to be their responsibility, they would prefer to share this responsibility with others. Some patient participants would prefer to share the responsibility of decision-making to avoid regret and blame for any adverse outcomes of their decision.

*“Yes, I always involve all the family members [in decision-making]: that would be the best [decision]. Like that, everyone will know, and everything will be clear. Otherwise, if something goes wrong later, I will be the one who is blamed (laughed).”* (YK3A: female patient (age 62) with stage III breast cancer, Christian, Javanese)

Other family caregiver participants believed it was their duty to decide on the patient’s care and would voluntarily fulfil that duty by taking up this role.

Likewise, some family caregiver participants considered it important to include more family members, as spreading responsibility over a group would make them less accountable than if they acted alone.

*“Everyone [in the family], everyone’s opinion [should be taken into account], not just one person’s. As we’re afraid that we’ll be blamed later on. So, it should be a majority vote, let’s say.”* (RSKD4B: daughter (age 35) of a patient with acute myeloid leukaemia, Muslim, Sundanese.)

As most patient and family caregiver participants saw it as the physicians’ responsibility to make the best recommendation, they would trust the physicians to make it and sometimes even to decide on their behalf.

*“Usually, we put our trust in the doctor, as that makes it simpler for us and [the doctor], as he/she is certainly more experienced [than us]”.* (RSKD4A: male patient (age 39) with stage 3A non-Hodgkin lymphoma, Catholic, Javanese)

### Decision-making in a state of dependency

One patient participant, who found it difficult to understand the complex medical information given by her physician, stated that she would rely on her children due to her self-perceived inability to process such information.

*“I’ll follow what my children say. The most important thing is that I follow [what they have decided for me] and [that I] prepare myself. That’s it. So, when the doctor asks for a discussion, I only listen – my children are the ones who ask more questions. I’m not smart enough [to understand the discussion]. Things were always explained, but I just couldn’t understand...”* (YK2A: female patient (age 53) with advanced non-Hodgkin lymphoma, Muslim, Javanese)

Some patient participants who lived with or were cared for by family members often felt dependent on them for decision-making.

*“To make decisions, our Dad always depends on us. He said, ‘As long as I’m being taken care of, I’ll follow [your decision].’ He also said, ‘Well, since I’m being taken care of by my children, I’m dependent on them’.”* (RSKD4B: daughter (age 35) of a patient with acute myeloid leukaemia, Muslim, Sundanese.)

#### Theme-4: complexities of future planning

Our study showed that two factors made it difficult for most participants to plan for the future: (a) their belief in God’s authority over life and (b) their difficulties in seeing the relevance of future planning.

### Belief in God’s authority over life

For participants who believe that God is the only one who can determine their fate in life, conversations on future care planning can be difficult, particularly those about the end-of-life phase. As these participants believed that they have to accept whatever God has planned for them or their family members, they viewed planning for future care to be irrelevant.

*“In my opinion, since we have faith, we are merely God’s creatures, [and we must remain certain that] everything has its written destiny. We certainly don’t know what will happen in future. But we just need to give up everything to God and to be sure that whatever is destined is best for us.”* (RSKD2A: male patient (age 36) with acute lymphocytic leukaemia, Muslim, Sundanese.)

Some participants also believed that thinking about death and dying would mean that they failed to think positively about God’s will.

*“We must believe that God has the best plan for everyone, whatever their condition. We don’t need to think negatively, especially not regarding God.”* (RSKD2B: husband (age 64) of a patient with stage II breast cancer, Catholic, Chinese.)

As many participants believed in the sanctity of life and their obligation to preserve one’s sacred life, they preferred to focus on making an effort to preserve life rather than thinking about and planning for adverse events.

*“No, I never think about that [i.e., possible bad scenarios]. I believe only in God, that humans must only make an effort, and that God is the one who will decide everything.”* (RSKD6A: female patient (age 54) with metastatic breast cancer, Muslim, Sundanese.)

These participants believed that before accepting and surrendering to God’s decision, they must first make their utmost effort to preserve sacred life.

*“For me, this [pursuing treatment] is one of our ikthiar [utmost effort]. According to Islam, we must first do ikhtiar, and after that, if anything bad happens to my husband, then it’s God’s will.”* (RSKD3B: wife (age 47) of a patient with stage IV lung cancer, Muslim, Minangkabau.)

### Difficulties in seeing the relevance of future planning

Some participants felt that it was not necessary to discuss future planning, as they believed that scenarios for the end-of-life phase were not relevant to the situation at the time of the interview.

*“Up till now, I’ve never thought about that, as I think a situation in which her vital organs fail, or something like that, may not happen. I’m still optimistic that the prediction is that she’s still going to be okay.”* (RSKD2B: husband (age 64) of a patient with stage II breast cancer, Catholic, Chinese.)

Similarly, participants who were unable to reflect upon the consequences of their advanced illness considered such planning unnecessary.

*“I always think positively [about my future], that I need to recover completely, be cancer free whatever it takes. I have to keep the spirit to recover and always think positively.”* (RSKD7A: female patient (age 30) with metastatic breast cancer, Muslim, Sundanese.)

Most participants preferred to keep a positive mindset. To spare themselves from the consequences to their mental wellbeing, they refrained from thinking about possible adverse events in the future.

*“Sometimes, I don’t want to think too much about this [end-of-life care preferences]. Not because I underestimate my illness, but sometimes I just don’t want to overthink it. I just wish for everything to go as it’s going now.”* (RSKD 4A: male patient (age 39) with stage 3A non-Hodgkin lymphoma, Catholic, Javanese.)

Other patient participants thought that they already had enough of a burden and that future planning should be done by family members.

*“In my opinion, patients with cancer already have quite a burden, so there’s no need to add to it with such questions [i.e., about preferences for future care]. Those can be asked to the family members.”* (RSKD9A: female patient (age 32) with acute myeloblastic leukaemia, Muslim, Javanese.)

To be able to plan for death, some family caregiver participants argued that one would first needs to be mentally ready.

*“Actually, it includes making a living will, right? In Islam, when we’re ready to face death, we should in fact make a living will. But it really depends on each individual. Although we’re Muslim, we’re not always ready to make living wills. Sometimes, we aren’t ready to face death.”* (RSKD3B: wife (age 47) of a patient with stage IV lung cancer, Muslim, Minangkabau.)

While discussing future decisions could be challenging for many of our participants, they were more open to discussing what mattered most to them, both in the moment and the future.

*“My wish for the future is not for myself but my family. I don’t want my condition to burden anyone else.”* (RSKD7A: female patient (age 30) with metastatic breast cancer, Muslim, Javanese.)

*“Yes, now, motivation, accompaniment, and spirituality are the most important for her.”* (YK4B: husband (age 51) of a patient with stage IV breast cancer, Catholic, Javanese.)

## Discussion

In this qualitative interview study on Indonesian patients’ and family caregivers’ perspectives on serious illness communication in oncology care, we found that four important factors influenced their engagement in serious illness communication. First, patients’ and family caregivers’ wish to be informed about the disease and its consequences depended on whether they perceived the information as important, relevant, or harmful. Patients and family caregivers alike tended to conceal ‘harmful’ information to protect their loved ones. Second, they wished bad news to be communicated empathetically and sensitively, particularly by using implicit words (euphemisms). Family caregivers found that mediating the delivery of bad news required a sensitive approach. Third, participants’ preferences for involvement in decision-making varied. Their preference for patient-centered, family-led or physician-led decision-making, was influenced by their ideas on patients’ rights, their perceived responsibilities, or patients’ state of dependency on others. Finally, most participants found future care planning to be challenging, due either to their religious beliefs, or to their difficulties in seeing its relevance for future care planning. Discussing what mattered most in the moment seemed more appropriate.

Our study indicates that different individuals appreciate different amounts of information about their illness and that information provision without careful consideration of patients’ preferences may disrespect patients’ values and religious beliefs. Although most patient participants reported that they wished to receive certain information on their illness (e.g., diagnosis, treatment options), many of them considered information on estimated life-expectancy harmful or irrelevant because of their religious beliefs. Available evidence shows that patients with cancer in general have various preferences for prognostic disclosure, with more people preferring broad indications of prognosis rather than concrete estimations [20]. More recent studies in Asia showed that open communication on prognosis might cause psychological distress or decrease patients’ quality of life [21–23]. Accordingly, an important first step before providing medical information to patients is to assess which information is preferred and could be helpful for patients.

Another important consideration regarding information provision was the cultural sensitivity of its delivery. Indonesia is known for its relatively high-context culture in which messages are not necessarily expressed explicitly but can be implied implicitly [7, 24–26]. For this reason, Indonesian healthcare professionals are often expected to convey a message gently while being sensitive to subtle non-verbal cues transmitted by their patients [7, 26]. Our study, as well as other studies among Asians [7, 27–29], indicate that euphemisms may facilitate communication with individuals who appreciate implicit communication. Additionally, our study showed that patients and family caregivers often see hope as an aspiration to fight illness and escape death, and thus consider communication that takes away such hope to be unempathetic. Healthcare professionals should be able to facilitate redefining of hope within the context of terminal illness by identifying short-term, realistic, and attainable goals [30, 31] while providing reassurance of non-abandonment [32].

Our study also identifies a cultural dilemma in which patients and family members alike tend to conceal harmful information, limiting the opportunity for their loved ones to be involved in further decision-making. Such common non-disclosure indicates the need for an approach that focuses more closely on the culturally related dilemmas of breaking bad news. Recently, the ARCHES framework (an acronym for Acknowledge concern, build Relationship, Common ground, Honour patient’s preferences, Emotional support, and supportive Solution) was developed [33]. This framework focuses on maintaining cooperative relationships with family, for example by showing sensitivity to family’s concerns, by finding shared goals, by ensuring the sensitive delivery of information to the patient, and, in order to uphold patient’s rights for information, by achieving consensus with the family on the best way forward [33]. Such initiatives, along with promoting honest communication between patient and their family members, could help overcome cultural barriers to information provision and advance care planning. Similar dilemmas may occur when engaging in advance care planning with Asian patients living in non-Asian countries. Therefore, further efforts to complement current Western-oriented curricula with communication strategies that address various cultural dilemmas is warranted.

Finally, our study showed that patients’ and family caregivers’ willingness to engage in advance care planning was affected by various beliefs about death and dying. Those who believed in God’s authority over life and their obligation to preserve their life would likely be less open to engaging in discussions about taking control of death or the withdrawal of life-sustaining treatments. Sufficient understanding of these beliefs could help practitioners determine whether and to what extent a patient could engage in advance care planning and how the conversation could be navigated while being respectful to patients’ beliefs. Our previous qualitative study of Indonesian healthcare professionals suggested that the transfer of sensitive medical information among religiously devout patients and family caregivers could be facilitated by circumspect conversation within their religious contexts [7]. For example, religious terminology such as “mudharah” (or harm) could help address the distant concept of “futile treatments” as the avoidance of greater mudharah [7]. Other studies in Western countries have shown the value of trained spiritual care providers in facilitating the exploration of patients’ values, goals, and preferences [34, 35]. Additionally, our study showed that advance care planning for future treatment can be very difficult for those who are not ready to engage in discussions of death and dying, or for those in a stable condition who cannot reflect on future deterioration. In such instances, exploration of patients’ values is one of the important goals of advance care planning, rather than merely focusing on eliciting patients’ preferences for future care, our findings.

### Strength and limitations

To the best of our knowledge, this is the first study to explore the perspectives of Indonesian cancer patients and their family caregivers on advance care planning. Due to the important role of family in Indonesia, we explored the perspectives of both these groups in order to disentangle the factors that play important roles in patients’ engagement in advance care planning. Based on our systematic reviews in Asia and consultation to a panel of Indonesian multidisciplinary experts, we developed an interview guide that enabled us to prompt culturally relevant questions. The robustness of our analysis was improved by using dual coders and triangulation by experts from various disciplines and cultural backgrounds.

When interpreting this study, two main limitations need to be considered. First, selection bias may have resulted from the fact that most of our participants had completed higher education and had been selected based on their willingness to participate in the study. This means that our findings may not be relevant for those with lower educational backgrounds and/or those who were not willing to participate in the study. Second, the interviewers’ background as healthcare professionals may have obscured participants’ responses during the interview. This risk was minimized by ensuring participants that any responses would not be disclosed to attending physicians and would not affect their care.

## Conclusions

Our study indicated that engagement in serious illness communication and advance care planning in Indonesia would be facilitated by several important factors, including culturally sensitive awareness of various perspectives on information provision, bad-news communication, decision-making, and future care planning. Advance care planning in Indonesia should address the importance of collective decision-making, religious beliefs, and the maintenance of social harmony, and should regard value exploration as its main goal. Further study is needed to explore the different perspectives of patients with various religious affiliations, races, and non-cancer life-limiting illnesses.

## Supplementary Information

**Additional file 1: Appendix 1.** Interview Guide (Patient). **Appendix 2.** Interview Guide (Family Caregiver). **Appendix 3**. Steps of thematic analysis and translation process.

## Data Availability

The original datasets generated and analyzed during the present study are not publicly available due to the requirement to preserve confidentiality, but are available from the corresponding author on reasonable request.
