# Supplementary material for: Advance care planning for patients with cancer and family caregivers in Indonesia: a qualitative study
Source: BMC Palliat Care. 2022 Nov 22;21:204. doi: 10.1186/s12904-022-01086-0 (PMC9682799; doi:10.1186/s12904-022-01086-0)
Supplement: Supplementary file 1 — Additional file 1: Appendix 1. Interview Guide (Patient). Appendix 2. Interview Guide (Family Caregiver). Appendix 3. Steps of thematic analysis and translation process. [file 12904_2022_1086_MOESM1_ESM.docx]

**Appendix 1**

**Interview Guide (Patient)**

1. The researcher introduces herself to the patient:
   1. To reduce the distance from the patient, introduce yourself as a researcher who is researching the process of discussing the topics in question.
   2. Explain that the patient’s information and the interview will be treated confidentially, that only the research team will have the access to the data, and that it will not affect patient’s treatment and care.
   3. Explain that participation in this study is entirely voluntary and will have no consequences for the care and management the patient receives at the hospital.
2. The researcher explains the study:
   1. The study is intended to explore the patient’s feelings and opinions about decision-making on their medical care and treatment: in the past, present, and future.
   2. The study is also intended to explore the patient’s feeling of uncertainty about the future, and how they deal with it.
3. The researcher explains the mechanism of the interviews:
   1. Duration: 45 to 60 minutes.
   2. Interviews will be recorded.
4. The researcher asks for the patient's approval to participate in the study and for their informed consent.
5. During the whole recording session, the researcher should try to avoid mentioning the patient’s identity (e.g., name, age, etc), for example by starting the recording with “This is interview number XX; today is Tuesday, 18^th^ July, 2019).”
6. The researcher asks the patient to talk about him/herself and to briefly explain their understanding of their current illness.
7. The researcher asks the patient to share about their experience of receiving information about their condition from their healthcare professional, and how they would have preferred it to be delivered.
8. The researcher creates connection with the patient, which can be done in several steps, as follows:
   1. Ask about the patient’s current situation: family, spouse, children; where they live; their current health condition, and their feelings about the current situation.
   2. Ask the patient how they feel about their illness.
   3. Ask the patient how they feel about their experience with medical decision-making.
9. The researcher asks the patient’s experience with medical decision-making about his/her current care and how they would have preferred it to be practiced.
10. The researcher asks what is important to the patient regarding their future, particularly if the disease progresses or their condition worsens.
11. The researcher asks what is important to the patient in terms of decision-making on future care:
    1. The researcher asks whether the patient is interested in or willing to think about their future and their future medical care.
    2. The researcher asks the patient’s opinion on who should make decisions regarding their future and their future medical care.
    3. The researcher asks the patient’s opinions on the future role of family in decision-making on the patient’s future medical care.
    4. The researcher asks the patient’s opinion on the physicians’ future role in decision-making about their future medical care.
12. The researcher asks whether the patient has prior experience of discussing a future care plan. The researcher allows the patient to share their experience, feelings, and thoughts on this.
13. During the interview process, the researcher will ask open questions to explore the patients’ understanding and views. He/she will encourage the patient to answer questions freely without trying to direct their answers.
14. The researcher will pay attention to and record the details of the conversation as well as the body language of the patient who are considered important during the interview.

**Appendix 2**

**Interview Guide (Family Caregiver)**

1. The researcher introduces him/herself to the family caregiver and explains the purpose of the study and the mechanism of the interviews. He/she asks the family caregiver’s approval for participating in the study.
2. If the family caregiver is willing to participate in the study, the researcher asks the family caregiver to sign an informed consent sheet.
3. The researcher asks the family caregiver's approval for recording the entire interview process and, if necessary, for noting important points during the interview.
4. The researcher asks the family caregiver to introduce him/herself, and to briefly explain his/her understanding of the illness of his/her family (patient).
5. The researcher asks what is important for the family caregiver regarding her/his family future care, particularly if the disease progresses or the patient’s condition worsens.
6. The researcher asks the family caregiver’s experience with medical decision-making for the care of their family with cancer and their opinions on the future role of the family in decision-making on the patient’s future care.
7. The researcher asks the family caregiver’s opinions on the future role of physicians in decision-making on the patient’s future care.
8. The researcher asks the family caregiver’s opinions on the extent to which the patient should be included in decision-making on his/her future care.
9. The researcher asks whether the family caregiver has had prior experience of discussing a future care plan. If the answer is yes, the researcher will allow the family caregiver to share their experiences, feelings, and thoughts.
10. During the interview process, the researcher will ask open questions to explore the family caregiver’s understanding and views. He/she will encourage the family caregiver to answer questions freely without trying to direct the caregiver’s answers.
11. The researcher will pay attention to and record the details of the conversation as well as the body language of the family caregiver.

**Appendix 3**

**
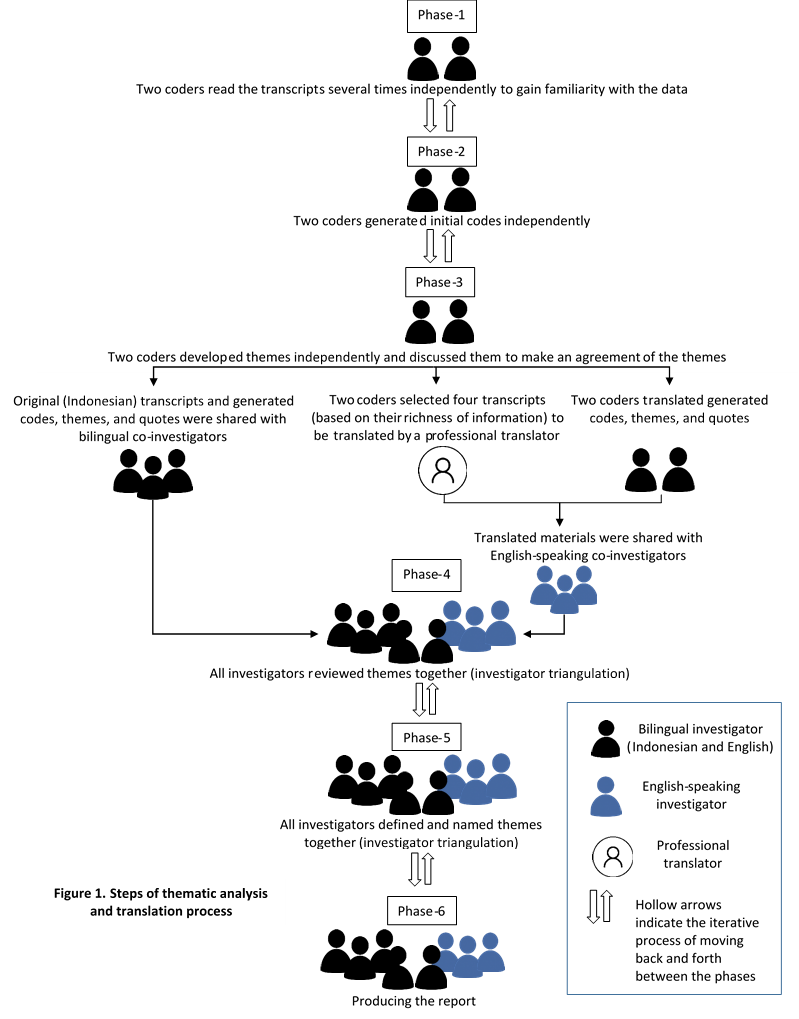
**
